# BrainSwarming, blockchain, and bioethics: applying Innovation Enhancing Techniques to healthcare and research

**DOI:** 10.1038/s41598-023-50232-y

**Published:** 2024-01-10

**Authors:** Anuraag A. Vazirani, Tony McCaffrey, Julian Savulescu, Sebastian Porsdam Mann

**Affiliations:** 1 Medical Sciences Division, University of Oxford, Oxford, UK; 2 Head, Computer Science Department, Eagle Hill School, Hardwick, MA USA; 3 Director & Chen Su Lan Centennial Professor in Medical Ethics, Centre for Biomedical Ethics, Yong Loo Lin School of Medicine, National University of Singapore, Singapore, Singapore; 4 Visiting Professiorial Fellow in Biomedical Ethics, Murdoch Childrens’ Research Institute, Royal Children’s Hospital, Melbourne, VIC Australia; 5 Oxford Uehiro Centre for Practical Ethics, University of Oxford, Oxford, UK; 6 Faculty of Law, University of Oxford, Oxford, UK

**Keywords:** Translational research, Medical ethics, Behavioural methods

## Abstract

Innovation in healthcare and biomedicine is in decline, yet there exist no widely-known alternatives to traditional brainstorming that can be employed for innovative idea generation. McCaffrey's Innovation Enhancing Techniques (IETs) were developed to enhance creative problem-solving by helping the solver to overcome common psychological obstacles to generating innovative ideas. These techniques were devised for engineering and design problems, which involve solving practical goals using physical materials. Healthcare and science problems however often involve solving abstract goals using intangible resources. Here we adapt two of McCaffrey’s IETs, BrainSwarming and the Generic Parts Technique, to effectively enhance idea generation for such problems. To demonstrate their potential, we apply these techniques to a case study involving the use of blockchain technologies to facilitate ethical goals in biomedicine, and successfully identify 100 potential solutions to this problem. Being simple to understand and easy to implement, these and other IETs have significant potential to improve innovation and idea generation in healthcare, scientific, and technological contexts. By catalysing idea generation in problem-solving, these techniques may be used to target the innovative stagnation currently facing the scientific world.

## Introduction

Brainstorming is a method for idea generation commonly employed across disciplines. Other methods to systematically increase the efficiency of idea generation have been explored in the fields of psychology and engineering. Much of this work has focussed on targeting insight problems—those whose solutions may require a change in approach, or a restructuring of the initial problem^1^. The Obscure Features Hypothesis posits that innovative solutions to a problem are based upon at least one previously ‘obscure’—novel or rarely noticed—feature of that problem^2^. However, many human habits, biases, and heuristics hinder the noticing of obscure features (Table 1). This line of research has led to the development of several Innovation-Enhancing Techniques (IETs) that assist in the identification of obscure features and so can be applied to enhance the ideation stage of solving engineering and design problems^3,4^.

**Table 1:** Known psychological obstacles to creative problem-solving.

Obstacle	Description	References
Functional fixedness	The habit of seeing an object for its designed use, and so being unable to use it in a new way	Duncker^5^
Design fixation	After seeing possible solution(s), future attempts to create an innovative solution are shaped by the solution(s) already seen: the solver’s own solutions resemble the solution seen	Jansson & Smith^6^
Goal fixedness	The solver stays close to the original phrasing of the problem, and so only considers certain kinds of solution	McCaffrey & Krishnamurty^4^
Analogy blindness	Difficulty adapting a solution from one area to another area	Gick & Holyoak^7,8^
Assumption blindness	The solver makes assumptions about the nature of the solution, and is unaware that those assumptions are being made	McCaffrey & Krishnamurty^4^

Given their origin in engineering contexts, these IETs have so far been used to target problems which require the use of tangible materials and resources (such as stone, bricks, and cement) to solve practical goals (such as building a bridge). However, many important problems in biomedical and scientific contexts concern goals that are conceptual or abstract in nature and may not necessarily be easily measurable, such as ‘benefitting patients’ or ‘decreasing health disparities’. Equally, such problems may require the use of intangible resources, such as software and data, that lack physical instantiation and are better characterised by their functions and affordances than their material composition.

Despite the frequent occurrence of problems in scientific research and healthcare requiring creative restructuring and insight, no studies to date have tested the efficacy of IETs to facilitate insight, innovation, or creativity in problems featuring intangible objects or conceptual goals. Enhancing creativity and idea generation in biomedicine has the potential to catalyse scientific and medical progress in highly cost-effective and efficient ways. This potential is particularly important at a time when research demonstrates a decreasing rate of innovation across scientific fields by multiple measures over several decades^9^.

We hypothesised that the IETs shown to enhance innovation and idea generation in engineering and design contexts can usefully be applied or adapted to problems featuring conceptual goals or intangible resources. We tested this by applying two IETs in parallel to a case study which involved identifying potential solutions to a problem involving a conceptual goal (furthering bioethical principles of beneficence, justice, autonomy, and non-maleficence) using an intangible resource (blockchain technology).

## Innovation-Enhancing Techniques

### BrainSwarming

**Figure 1:**
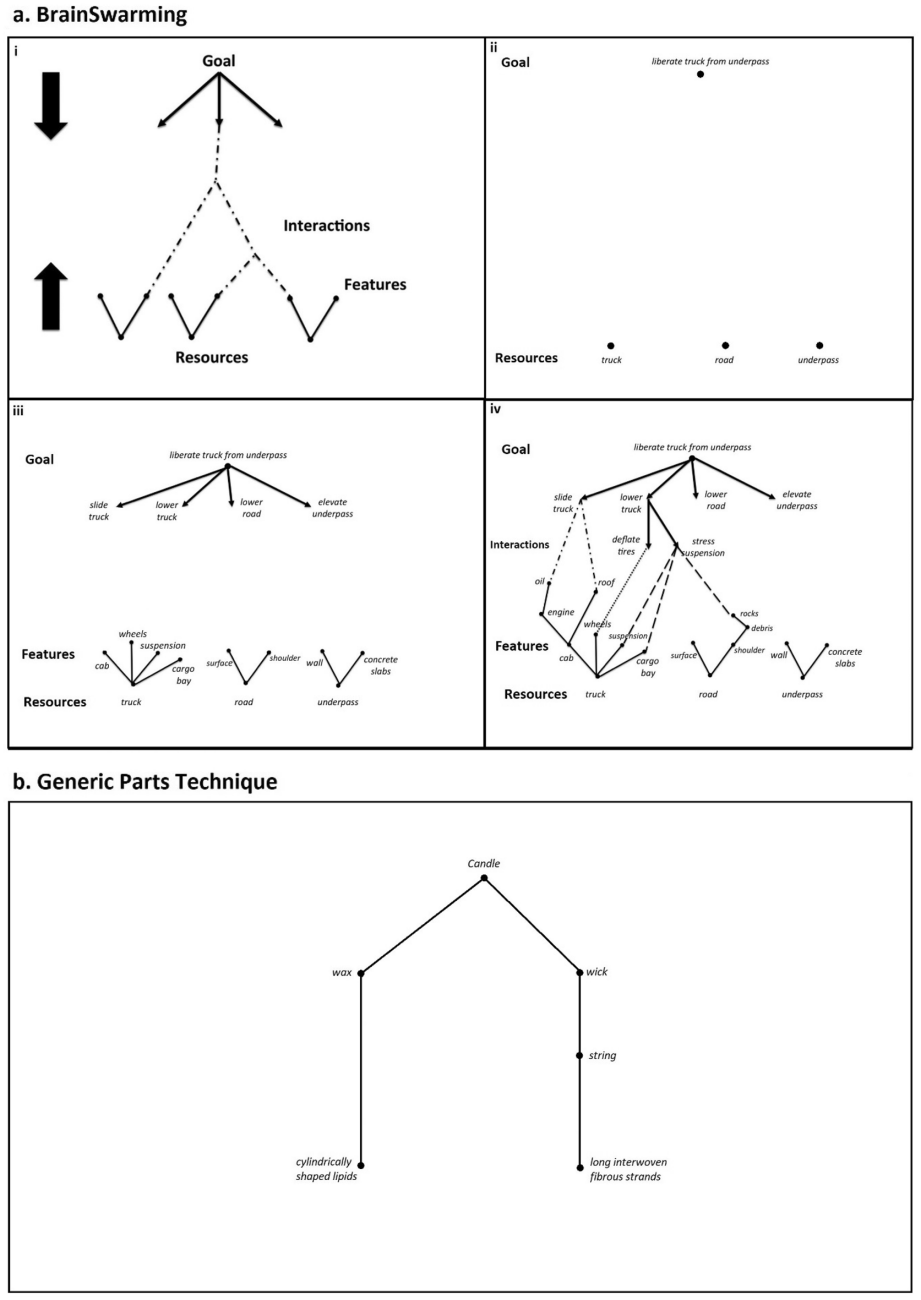
Innovation-Enhancing Techniques. (**a**) BrainSwarming: (i) In this example, we use the *Stuck Truck* problem. A delivery truck was too tall for an underpass, and becomes wedged tightly beneath it. Without further damaging the truck or the underpass, and without assistance from others, how can the driver get the truck unstuck? (ii) The main goal (*‘liberate truck from underpass’*) is placed at the top of the diagram and is broken down into refined goals that grow downwards (*e.g. ‘slide truck’, ‘lower truck’, *etc*.*). (iii) Known resources (*e.g. ‘truck’, ‘road’, ‘underpass’*) are placed across the bottom of the diagram and are broken into features and parts, which grow upwards as solid lines. (iv) The resources and goals are interacted together, signified by dotted lines, to produce effects that help satisfy the refined goals and ultimately the top goal. Where the two directions meet, a candidate solution emerges. For example, if you stress the suspension with available heavy objects (*e.g. rocks*), you can lower the truck and possibly free the truck from the underpass. (**b**) Generic Parts Technique: Suppose we need to tie two things together and we have only a candle. Applying the Generic Parts Technique, the candle’s composition is described as consisting of wax and a wick. In this context, the descriptor ‘wick’ is associated with burning to emit light. A more generic description is ‘string’, which is closely associated with tying things together. Removing the wax to free the string gives us something to use for tying. For completeness, one even more generic description of a string with smaller parts is *‘long interwoven fibrous strands’*.

BrainSwarming graphs (see Fig. 1a for a worked example), originally known as bidirectional networks (bi-nets)^10^, were designed as a means of visualising problem solving and facilitating simultaneous idea generation in a problem-solving group^3^—where social dynamics, such as having to wait turns, or certain individuals dominating conversation, may hinder progress.

In a BrainSwarming session, a short description of the problem to be solved, the goal, is placed at the top of a two-dimensional graph on any medium that allows adaptation and visualisation, such as digital mind-mapping software, a whiteboard, or a large sheet of paper. Resources available to solve the problem are placed at the bottom of the graph.

Next, the goal is iteratively refined downwards by placing more detailed or nuanced expressions of the same goal underneath it (here referred to as ‘refined goals’). Similarly, resources are iteratively refined upwards into their parts and components.

Finally, where a refined goal and resource could together form a solution a link is created between them. Goals and resources thus form networks that ultimately converge in interactions between resources and refined goals, each representing a potential solution to the problem.

In this way, BrainSwarming allows for the visualisation of both top-down problem framing and bottom-up problem solving^10^. Pilot studies demonstrate an increased rate of idea-generation in less time for individuals performing BrainSwarming compared to traditional brainstorming (115 ideas in 15 min vs. 100 ideas in 60 min)^3^. While the speed and volume of ideas generated may not in itself reflect the quality of these ideas, they are important factors in any creative process. Other things being equal, a technique that improves these factors will translate into speedier progress towards the solution of goals.

### Generic Parts Technique

The Generic-Parts Technique (GPT) (see Fig. 1b for a worked example) is a different IET designed to supply new information or to help re-interpret existing information about the resources involved in a creative or design task, by enabling the noticing of obscure features through decomposition and redescription. Used in conjunction with BrainSwarming, the GPT provides a systematic method of refining the resources placed on the bottom half of the graph.

The GPT involves a two-step iterative process of refinement, at each stage of which the following questions are asked of a resource object:

1. Can this object be broken-down further? If so, the problem solver should decompose the resource into its components and place these on a new leaf in a hierarchical diagram;
2. Does this description imply a use? If so, the solver should reframe the resource description neutrally to prevent functional fixedness following from use descriptions (see Table 1).

In a test on eight insight problems, human subjects trained to use the GPT method reached a solution 67.4% more often than controls, (Cohen’s d = 1.59—a large effect size)^2^.

## Methods

Our aims were:

1. To evaluate whether IETs can usefully be applied to identify potential solutions to problems involving (a) conceptual goals, and (b) intangible materials as opposed to material objects.
2. To use these techniques to identify specific innovative applications of blockchain technologies in biomedicine that improve ethical outcomes. We aimed to identify 100 such potential applications.

### Goals and Resources for BrainSwarming graph

We began by populating our BrainSwarming graph with our chosen ultimate conceptual goal (furthering ethical goals in biomedicine), and our initially defined resources (blockchain technology).

*Population of refined goals*

Having chosen our conceptual goal of furthering bioethics, we defined our refined goals as corresponding to the four classical principles of biomedical ethics as described in the classic textbook by Beauchamp & Childress^11^ (Table 2). We further broke down these principles as much as possible following the analyses by Beauchamp and Childress themselves. Where necessary, we complemented these with our own conceptual analysis to create more detailed levels of refined goals. Thus, it bears emphasis that others carrying out the same exercise would likely choose other refinements; however, for the purposes of applying innovation-enhancing techniques, we considered this essentially pragmatic approach to be sufficient.

**Table 2:** Bioethical principles as goals.

Principle	Explanation	Examples of biomedical policy enacting this principle
Beneficence	The ethical desirability or ideal of benefitting people. The goal is, broadly speaking, to improve people’s welfare, help them achieve their goals, satisfy their preferences, and to avoid harm and frustration	Facilitating biomedical research Improving access to and quality of medical treatment Reducing costs and barriers associated with access to research and practice
Non-maleficence	This principle appeals to the idea that, in addition to benefitting people, there is a separate duty or ideal not to cause them harm, or frustrate their desires, satisfactions, or goals. This is famously summed up in the Hippocratic Oath as ‘first, do no harm’	IRB/Ethics review Policy of not communicating unactionable incidental findings High standards for safety of devices used in biomedical research and practice
Justice	A separate set of concerns about the distribution of benefits and harms, the need for fairness in policy, experimentation, and practice, and the observation of the rule of law and relevant legislation	Demographically representative sampling in biomedical studies Laws against discrimination Reporting conflicts of interest
Autonomy	The ideal of respecting people’s choices regarding their own life and actions	Informed consent Research informed by patient advocates Confidentiality

*Population of resources*

In order to further populate the Resources (bottom) section of our BrainSwarming graph, we broke down blockchain technology (a type of cryptographic technology used principally to store records) into its necessary components and features (IT artefacts): Immutable Audit Trail, Consensus Mechanism, Encryption Mechanism, Distributed Ledger, and Smart Contracts, as described in the literature on blockchain affordances^12^ (Table 3).

**Table 3:** IT Artefacts of Blockchain as resources.

Artefact	Explanation
Immutable Audit Trail	Every block in the chain is created using a hash of the previous block. A change in the content of a previous block would affect all subsequent blocks’ hash values, exposing any attempt at tampering. This preserves integrity of data stored in the chain
Consensus Mechanism	A way of ensuring each node contains an identical copy of blockchain data, and agrees on any additions
Encryption Mechanism	Asymmetric key cryptography: each entity interacting with a blockchain is issued two unique identifiers (keys)—a public key serving as a public address, and a private key serving as a password or signature, to prove authenticity
Distributed Ledger	A store of information, distributed to all nodes in the network
Smart Contracts	An algorithm (program) which is executed automatically when certain pre-defined criteria are satisfied

### Application of Generic Parts Technique to intangible resources

In order to refine our resources beyond the IT artefacts of Blockchain, we applied the Generic Parts Technique to each IT artefact following the two-step iterative process described above. We used our results to continue to populate the Resources section of our BrainSwarming graph. Where it was not possible to decompose a resource further by decomposing words (i.e. ‘audit trail’ into ‘audit’ and ‘trail’), we used a dictionary definition for that word in order to decompose the resource (e.g. ‘contract’ into ‘enforceable’ and ‘agreement’), choosing the definition germane to the context where relevant. When unable to complete this process, for example because use of a definition did not permit decomposition, the etymology of the word was used to refine the resource (e.g. ‘encryption’ into ‘hidden’ and ‘inside’).

### Application of BrainSwarming to conceptual goals and intangible resources

Having fully defined our refined goals and broken down our resources into their component parts, and used these to populate our BrainSwarming graph, we began searching for solution paths connecting the upward vectors of component resources with the downward vectors representing refined goals. We identified 100 potential solutions and marked these with connecting lines.

## Results

### Case study: facilitating bioethical goals using Blockchain technology

**Figure 2:**
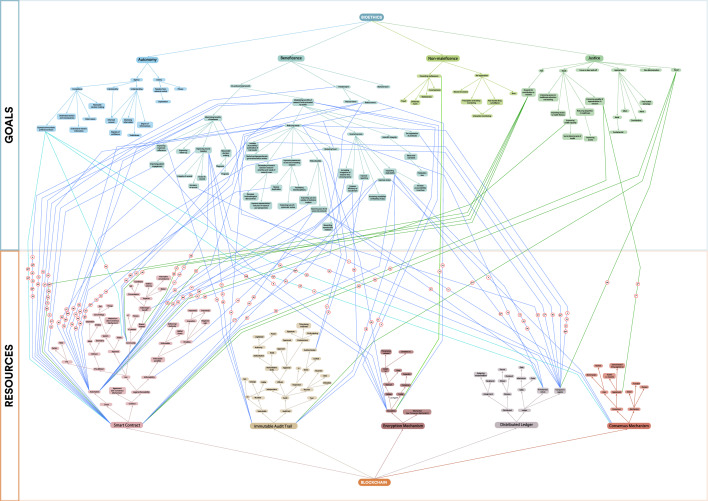
Application of Innovation Enhancing Techniques to a problem involving intangible goals and resources. BrainSwarm demonstrating 100 possible use cases of Blockchain technology to further ethical goals in healthcare and research. Numbering relates to possible use cases, expanded in Extended Data Table 1. * indicates the use case is highlighted and expanded in Table 4.

Applying BrainSwarming and the GPT to our case study, we generated 100 solution paths representing potential uses of blockchain technologies to further ethical objectives in clinical and research contexts (Fig. 2, Extended Data Table 1), thereby demonstrating the efficacy of the application of these techniques to the novel context of conceptual goals and intangible resources. For reasons of space, we highlight 25 of these potential solutions alongside their respective BrainSwarm solution pathways in Table 4 and provide more detailed explanations for five of these below. For ease of analysis and presentation, we further classified our solution pathways into ‘use concepts’—groups of solutions falling into thematic groups (Table 5). Several solutions were reached via multiple routes on the BrainSwarming graph—demonstrating the potential of these particular use cases to satisfy more than one bioethical goal.

**Table 4:** 25 solution pathways identified by BrainSwarming to achieve ethical goals in biomedicine using blockchain technology.

a	Refined goal	Refined resource	Description of potential use case
1	Increase efficiency of recruitment	Advantage/benefit (+ Smart contract)	Token payment for clinical trial goals eg. recruitment, replication, pre-registration, appropriate reporting
3	Reducing fraud	Auditor/looker + encryption	Verified pseudonymous governance reporting system for health service employees (including 'whistleblowing')
6	Improving equality of representation of research users	Consensus/agreement	Individuals with a condition of interest pseudonymously and verifiably share information about their condition to assist research, with option for tokenised or other payment; may involve use of NFT for personal data sets
	Express/communicate preference/choice	Transparent register	
	Maximizing benefits of research	Smart contract	
7	Improving equality of representation of research users	Consensus/agreement	Tamper-proof system to enable pseudonymous voting on research priorities according to encoded rules—may include voting restrictions or permissions, and preferential weighting
11	Improving review	Enforceability	Smart contract-enforced protocols for funders, authors, reviewers, and others
13	Express/communicate preference/choice	Transparent register	Advanced directives stored as blockchain hashes to prevent tampering
17	Pre-registration of protocols	Transparent register	On-chain timestamped protocol registration; may include token incentivisation to register
	Improving use of and access to protocols	Transparent register	
20	Express/communicate preference/choice	Smart contract	Informed consent—consent given, but automatically revoked if certain conditions are met. ['Practical Implementation of Consent']
21	Improving patient engagement	Smart contract	Gamification: token payments for treatment adherence
	Improving treatment adherence	Smart contract	
30	Rewards for contributions to research	Smart contract	Incentivising healthcare professionals to develop innovations and improvements to clinical practise by allowing them to share in savings arising from those innovations and improvements, through smart contracts
33	Reducing error	Automation	Automatic flagging of drugs or devices found to be outdated, sub-standard, harmful, wasteful, etc.; with suggestions for alternatives. To include the equivalent of Field Safety Notices
38	Reducing waste	Community	DAO for hospital governance or clinical management, with option for pseudonymous input (eg voting) from relevant stakeholders
40	Maximizing benefits of research	Smart contract	Blockchain used to automate or manage innovation enhancing tools, such as a BrainSwarming tool; allowing pseudonymised editing (including to refinements, goals, and creation of new graphs); with embedded AI technology to suggest analogues; may include award of tokens for effective solution paths
	Maximizing benefits of treatment	Automation	
43	Express/communicate preference/choice	Smart contract	DAO managed funding pool for innovative startups—funders buy tokens and vote on proposals, winner(s) by vote receive funding; funders receive proportional share of IP
48	Maximizing benefits of research	Smart contract	Tokens in place of grant funding; use restricted to governance, research, or other relevant costs
59	Desert	Immutable audit trail	Minting NFTs that represent ownership, which can be traded or fractionalised; complement or alternative to patent system
60	Express/communicate preference/choice	Consensus mechanism	DAO-based voting as a method of determining scientific consensus
68	Reducing waste	Transparent register	Labour exchange for medical or research staffing, including option for certifications, ratings, and pseudonymity. Individuals prosent depending on factors such as salary; exchange is automatically updated and distributed
70	Express/communicate preference/choice	Transparent register	Practical implementation of meta-consent
72	Improving record keeping	Automation	Gamification: token payments for healthy lifestyle / behaviours
	Prevent harm	Smart contract	
73	Rewards for contributions to research	Smart contract	Micropayments to authors / scholars when their work is accessed or viewed
76	Maximizing benefits of treatment	Automation	Real-time update of data in online publications, may include journal articles
92	Improving replicability	Timestamping	Timestamped verified snapshot of data at specific or random stage of research, automatically delivered to pre-specified interested parties eg. funders
95	Reducing disproportionate governance/ethical review	Transparent register	IRB decisions stored on blockchain, decision relating to paper made public on paper acceptance/publication
99	Reducing fraud	Automation	Automated checks on suggested reviewers to exclude those eg. with known conflicts, from same organisation

**Table 5:** Solution pathways classified into use concepts—groups of solutions with a common theme.

Use concept	
Internet of Things	21
Enforcing trial protocols	11, 76, 92, 95
Enforcing rules	13, 33, 99
Gamification / incentivisation	1, 6, 21, 30, 40, 72, 73
Other token use	6, 7, 59
Financing	43, 74
Supply chain management	68
Administration / governance	1, 7, 11, 30, 33, 60, 68, 92, 95
Consent	13, 20, 70
Prosent & knowledge provenance	6, 59, 68
Voting & Consensus	7, 43, 60
Pseudonymous verification	6, 7, 40, 68, 70
Data & knowledge verification	13, 76, 92, 95, 99

### Examples of solution pathways

Below we outline five separate and distinct examples of solution pathways identified during our BrainSwarming process. We present these solution pathways as evidence that the application of IETs to contexts involving conceptual goals and intangible resources is feasible, and can indeed lead to effective identification of potential solutions to the specified problem. As with any other method in innovation, solutions identified by BrainSwarming, which pertains principally to the idea generation stage of the innovation process, must still be subsequently and rigorously evaluated according to their own merits before ultimately being implemented. We briefly explain each of the five example solution pathways below, refraining from detailed normative, technical, economic, or political assessment of the kind that would be necessary to complete the process of developing and implementing an innovation.

*1. Conditional informed consent*

Informed consent is a cornerstone of everyday clinical and research practice. However, it is often seen as a burden, overlooked, or implemented in ways that might shield a research project from legal liability but do little to respect the ideal of fully informed consent. In particular, many current consent procedures are largely static (patients may always withdraw from studies but otherwise their preferences cannot easily be updated) and unconditional (patients either consent or they do not).

Smart contracts are programs that execute on data contained in blockchains when specific conditions are met (e.g. ‘transfer payment sum if and only if title deeds are uploaded to property register’). If connected to a data oracle such that the relevant information is available to a blockchain-based smart contract, consents stored on a blockchain could be made conditional by automatically revoking consent should certain conditions arise. For example, consent for data use might be given for a fixed period or for certain uses only, or by certain individuals or groups. In addition to automaticity, a blockchain-based implementation of this use case would be transparent and tamper-proof.

*2. Blockchain managed advanced directives*

Advanced directives, also known as living wills, are documents expressing a person’s preferences towards future medical treatment and research participation decisions in the event of cognitive or other incapacity. Advanced directives suffer from several practical problems, including difficulty in accessing, verifying, and dating them, as well as the issues identified above relating to consent in general, such as being hard to update, unconditional, or insufficiently detailed or specific. Though issues relating to verification and dating can be addressed using professional services such as notarisation, these can be costly and time consuming. Dating advanced directives is of great importance, in part to assess whether they were made during a period of capacity but also because later versions of advanced directives are supposed to override earlier ones.

A possible solution to these issues, identified by our BrainSwarm, is to submit hashes of advanced directives to a blockchain-based registry. This has the potential to address issues of cost and effort (due to automation and removing the need for a notary, other witness, or lawyer), access (hashes of, but not the advanced directives themselves, would be publicly available on-chain), and ascertaining time (through the timestamping function of blockchains).

*3. Tokenized incentives for treatment adherence/healthy behaviors*

One of the most effective means of improving treatment adherence and healthy behaviour is through cash incentives^13,14^. However, these programs are not widely used in part due to ethical concerns over fairness, trade-offs, and opportunity costs^12,15^.

A potentially novel instantiation of this idea would be to reward treatment adherence and healthy behaviours with cryptocurrency tokens. A government could issue such tokens directly to individuals or through an intermediary and could imbue them with value by allowing them to be used, for example, for tax payments or for other government fees. Such a scheme would have the potential to reap the benefits of cash payments while obviating some of the associated ethical concerns, notably surrounding trade-offs and opportunity costs, as tokens would be free to mint and would not detract from other state health expenditures. The scheme could be set up to be financially self-sustaining by ensuring that payments made are outweighed by the overall money saved through improved population health.

*4. Smart contract-based checklists for clinical trials*

A significant proportion of biomedical research suffers from methodological flaws and a lack of statistical power^16^. Institutional and ethical review boards are supposed to review scientific merit in addition to legal compliance and ethical acceptability^17^ and are well-positioned to do so, since most human subject research has to undergo such an ethical review process. In practice, there is significant variability in the extent to which review boards attempt and are successful at ensuring scientific merit^16,18^.

Checklists have long been used to increase consistency and reduce errors in safety–critical contexts such as aviation and are increasingly applied in medicine and surgery^19^. A potentially novel application of this identified in our BrainSwarm is a smart contract-based checklist for institutional and ethical review. IRB members would fill out a review checklist on a hypothetical web portal. Progression through stages of protocol review would be locked by smart contract and predicated on submission of each section of the template. Upon submission, responses would be encrypted and sent to a repository via smart contract, which would also timestamp the submission. These responses could then be subject to random or automated audits.

The automated nature would alleviate potential concerns of ‘audit creep’ as it would not involve additional labour for IRBs. Timestamping could be used to document review process steps being taken in reasonable timeframes and in the correct sequence—not only providing incontrovertible evidence in cases of discrepancies or disputes, but also likely increasing the transparency of and trust placed in IRB review processes. Information on thoroughness and speed of reviews could also be used internally for quality improvement.

While the progress lock may be configured to force an IRB to make some kind of statement about the methodological merits or otherwise of proposed research, it is not intended to obviate or replace current protocols and legal frameworks, but rather to augment their implementation. Given the fundamental importance of basic scientific merit checks for overall scientific progress, however, any innovation which leads to improvements in this process would be worth weighing against these concerns. Other methods to develop the quality of proposals prior to submission should also continue to be utilised.

*5. Ethical approvals released with published studies*

Institutional and ethical review board decisions demonstrate a large degree of variability in the interpretation of regulations, value judgments, level of review required (full, none, or expedited), time to reach a decision, and quality of reasoning between different review boards^17,20,21^. Increasing transparency and accountability of ethical and institutional review, for example via publishing IRB decisions, has been proposed as a means of addressing these issues^22^. Such calls are sometimes resisted on the basis that increased transparency would be expensive and risks making public confidential information^23^.

A potentially novel means of addressing these concerns would be possible if ethics and institutional review are implemented on-chain, as outlined above. A smart contract could monitor trial publications and automatically decrypt and selectively publish review decisions relating to successfully published trials. The automated nature of this process would address expense concerns, while conditioning release of reviews on successful publication would partially address confidentiality concerns (as much of the potentially confidential information would be published anyway in the associated research paper) as well as contributing to transparency.

While separate confidentiality concerns related to IRB meeting minutes, memos, and other internal documents (rather than research protocols or participant data) are not addressed by this proposal, the fundamental role of the ethical review process in facilitating or inhibiting scientific progress makes any improvement in process, however partial, equally fundamental. These separate concerns could be addressed by having a specific form of review intended for publication alongside successful projects, which includes key information on ethics and methods reasoning but not more IRB-specific information such as meeting notes.

## Discussion

We set out to test whether BrainSwarming and the GPT could be adapted for use in biomedical contexts, involving conceptual, and in our case normative, goals, and software-based intangible resources. We generated 100 possible solution paths using only a small proportion of the nodes on the BrainSwarm, demonstrating the applicability of these tools in this novel context. Some of our ideas appeared more than once on our BrainSwarming graph as connections between multiple nodes. Combining blockchain-enabled prosent requests (Porsdam Mann et al. 2020) with token payments, for example, was identified at various points as a solution path between the goals of autonomy, beneficence, and justice and smart contracts, consensus, and transparent register resources. We considered duplication a promising sign indicating the potential of these use cases to satisfy more than one goal, or to be repurposed for multiple goals.

In adapting BrainSwarming and the GPT to normative goals and blockchain resources, we necessarily made operational choices which have influenced the solutions we defined. For example, to refine our normative goals, we needed to disambiguate and define abstract ethical concepts. We chose a pragmatic way forward by basing our initial analysis of these concepts on their canonical description in Principles of Biomedical Ethics by Beauchamp & Childress and supplemented these with our own understanding of the concepts involved. This is a notoriously difficult and controversial task, and others repeating the exercise may well have chosen differently. While other principles or approaches to biomedical ethics may have been equally valid and useful, we considered our choice justified given the simplicity and widespread use of these principles in bioethical scholarship and practice. The refined goals in our BrainSwarm could for example have been defined to include the 15 principles described in UNESCO’s Universal Declaration of Bioethics and Human Rights, or to include the United Nations Sustainable Development Goals, although our approach here was sufficient for us to discover 100 solution pathways. Importantly, our aim of evaluating the usefulness of IETs was independent of agreement concerning our analyses and refinements of bioethical principles. Indeed, the ability of others to iteratively refine these concepts in different ways to the ones employed here may well be a strength of such techniques, in that it may allow for the classification and description of goals and resources in ways that lead to the identification of further potential use cases.

To refine our resources and so populate the ‘Resources’ section of our BrainSwarm, we applied the GPT to each blockchain component (‘IT artefact’). We note that this is just one possible way to apply the GPT to intangible tools: our goal in doing so was to describe aspects of our available resources in ways which highlight potentially useful facets thereof, rather than to provide a canonical partition of these features, which is unnecessary for the purposes of applying the IETs described here. Thus, colleagues carrying out a similar exercise may arrive at different definitions and component resources, and so other innovative solutions to the problem.

A similar point applies not only to our breakdown of goals and resources but to the potential solutions we identified. Many of our identified solution pathways will be innovative, and potentially useful to clinical and research practice. As IETs are methods in idea generation and innovation, the solutions reached by a different group of individuals would differ from ours. Their usefulness and novelty will necessarily be influenced by the degrees of expertise of those carrying out the exercise.

Of note, solution paths were identified at an unpredictable rate. It was not the case, as might have been expected, that the first few hours invested lead to disproportionate numbers of potential solution paths. Had we not chosen an arbitrary cut-off of 100 potential use cases, we suspect we would have been able to identify many more solution paths at deeper levels of node hierarchy.

Finally, it should be noted that we deliberately chose normative goals and intangible resources at a high level of abstraction to test our hypotheses. This choice was motivated by the reasoning that if we were successful in repurposing the Generic Parts and BrainSwarming techniques to maximally abstract and intangible goals and resources, these techniques are also likely to be applicable to less abstract goals and less intangible resources.

## Conclusions

BrainSwarming and GPT were successful in helping us to discover innovative solutions to the abstract problem we chose. Our experience leads us to conclude that these innovation-enhancing techniques can usefully be applied and adapted to clinical and research contexts. We demonstrated their potential by applying them to a case study involving the use of blockchain technologies to facilitate ethical goals in biomedicine. Many of the solutions identified are novel, though they necessarily reflect our knowledge and skill sets.

The vast potential of IETs in healthcare and research is highlighted by the fact that others with different background experience taking slightly different approaches towards adaptation of these techniques may come up with different but equally innovative solutions to the same problem.

### Supplementary Information

Supplementary Information.

## Data Availability

All data generated or analysed during this study are included in this article and its supplementary information files. It should be noted that, because of the idiosyncratic nature of idea generation and innovation, replication of the study may lead to different, but equally valid results.
