# Supplementary material for: BrainSwarming, blockchain, and bioethics: applying Innovation Enhancing Techniques to healthcare and research
Source: Sci Rep. 2024 Jan 10;14:832. doi: 10.1038/s41598-023-50232-y (PMC10781689; doi:10.1038/s41598-023-50232-y)
Supplement: Supplementary file 1 — Supplementary Information. [file 41598_2023_50232_MOESM1_ESM.docx]

Extended Data Table 1: 100 potential use cases for blockchain technologies for advancing bioethical principles in healthcare and research, identified through our BrainSwarm. ‘Refined Goal’ and ‘Sub-resource’ columns indicate terminals of solution paths between goals and resources respectively. Multiple listings for the same use case indicate that it was identified by multiple solution paths, thus suggesting potential to address multiple goals.

|  | **Refined Goal** | **Sub-resource** | **Description of potential use case** |  |
| --- | --- | --- | --- | --- |
| 1 | Increase efficiency of recruitment | Advantage/benefit (+ Smart contract) | Token payment for clinical trial goals eg. recruitment, replication, pre-registration, appropriate reporting. | * |
| 2 | Reducing bias | Auditor/looker + encryption | Verified pseudonymous voting for IRB or other groups potentially subject to bias. |  |
| 3 | Reducing fraud | Auditor/looker + encryption | Verified pseudonymous governance reporting system for health service employees (including 'whistleblowing'). | * |
| 4 | Improve review | Consensus mechanism | Verified pseudonymous system for crowd-sourced peer review. |  |
| 5 | Improving accuracy | Consensus mechanism | Crowdsourced expertise - Verified pseudonymous system to obtain expert opinion (eg. medical second opinion, research question with lack of evidence). |  |
|  | Reducing waste | Transparent register |  |  |
| 6 | Improving equality of representation of research users | Consensus/agreement | Individuals with a condition of interest pseudonymously and verifiably share information about their condition to assist research, with option for tokenised or other payment; may involve use of NFT for personal data sets. | * |
|  | Express/communicate preference/choice | Transparent register |  |  |
|  | Maximizing benefits of research | Smart contract |  |  |
| 7 | Improving equality of representation of research users | Consensus/agreement | Tamper-proof system to enable pseudonymous voting on research priorities according to encoded rules - may include voting restrictions or permissions, and preferential weighting. | * |
| 8 | Improving patient engagement | Encryption | Pseudonymous communication with healthcare professional or clinical trial researcher. |  |
| 9 | Maximizing benefits of research | Encryption | Mechanism allowing individuals to contribute data pseudonymously under certain predefined circumstances in return for a token payment; enabling researcher to ensure diversity of dataset and representative sample. |  |
| 10 | Preventing malfeasance | Encryption | Reporting system for patients suffering from abuse, limiting identity access. |  |
| 11 | Improving review | Enforceability | Smart-contract enforced protocols for funders, authors, reviewers, and others. | * |
| 12 | Improving treatment adherence | Immutable audit trail | Tamper-proof proof of location, eg. for psychiatric treatment / prisoners. |  |
| 13 | Express/communicate preference/choice | Transparent register | Advanced directives stored as Blockchain hashes to prevent tampering. | * |
| 14 | Reducing waste | Immutable audit trail | Supply-side auditing of stocks - eg. tests, reagents, supplies. |  |
| 15 | Improving replicability | Transparent register | Signing off on specific part of methodology (eg. data analysis) to indicate assumption of responsibility. |  |
| 16 | Reducing waste | Timestamping | Supply chain management - Stock level monitoring and automated re-ordering and payment when stocks run low - eg. tests, reagents, gloves. |  |
|  | Reducing error | Automation |  |  |
|  | Reducing waste | Transparent register |  |  |
| 17 | Pre-registration of protocols | Transparent register | On-chain timestamped protocol registration; may include token incentivisation to register. | * |
|  | Improving use of and access to protocols | Transparent register |  |  |
| 18 | Improving replicability | Immutable audit trail | Automated data upload from Internet of things (incl. machines and wearables) and hash verification. |  |
| 19 | Reducing error | Penalties | Automated task execution if device indicates faultiness, eg. warning or fine. |  |
| 20 | Express/communicate preference/choice | Smart contract | Informed consent - consent given, but automatically revoked if certain conditions are met. ['Practical Implementation of Consent'] | * |
| 21 | Improving patient engagement | Smart contract | Gamification: token payments for treatment adherence. | * |
|  | Improving treatment adherence | Smart contract |  |  |
| 22 | Improving patient engagement | Smart contract | Token payments to those who enrol in clinical trials. |  |
| 23 | Improving reporting | Automation | Reminder to adhere to reporting guidelines when deficient; may include penalty/reward. |  |
| 24 | Maximizing benefits of treatment | Automation | Emergency notification triggering, eg. for first responders. |  |
| 25 | Improving use and validity of statistical analysis | Automation | Automated verifiable statistical analysis for research, may be of IoT data |  |
|  | Improving use of and access to protocols | Automation |  |  |
| 26 | Increase accessibility/publication of trial results | Automation | Automated follow-up for results of registered but unreported trials |  |
| 27 | Increase efficiency of recruitment | Automation | Automating the process for obtaining consent for use of trial participant data in future studies. |  |
| 28 | Increase efficiency of recruitment | Automation | Automated contact or reminders to patients, eg. for annual check ups, screening. |  |
| 29 | Increasing integration of research into clinical practice | Automation | Automatic updating of systematic reviews and meta-analyses. |  |
| 30 | Rewards for contributions to research | Smart contract | Incentivising healthcare professionals to develop innovations and improvements to clinical practise by allowing them to share in savings arising from those innovations and improvements, through smart contracts. | * |
| 31 | Improving health equality | Automation | Smart contract to automatically check eligibility for unclaimed social entitlements. |  |
| 32 | Reducing error | Automation | Pharmaceutical supply chain management - including automated ordering and dispensing, with immutable audit trail. |  |
| 33 | Reducing error | Automation | Automatic flagging of drugs or devices found to be outdated, sub-standard, harmful, wasteful, etc; with suggestions for alternatives. To include the equivalent of Field Safety Notices. | * |
| 34 | Express/communicate preference/choice | Consensus mechanism | Crowd-sourced research funding, implemented by a DAO. |  |
| 35 | Reducing error | Automation | Automated payment of salaries, bills, and grants. |  |
| 36 | Reducing waste | Automation | DAO to improve efficiency of administration of research (such as literature search) (including option for citizen science) and healthcare processes, such as funding. |  |
|  | Rewards for contributions to research | Smart contract |  |  |
|  | Maximizing benefits of research | Automation |  |  |
| 37 | Reducing error | Smart contract | Bug Bounty - Token payments for spotting and/or fixing problems eg. in medical devices or hospital equipment. |  |
| 38 | Reducing waste | Community | DAO for hospital governance or clinical management, with option for pseudonymous input (eg voting) from relevant stakeholders. | * |
| 39 | Rewards for contributions to research | Smart contract | Blockchain tokens used to vote for and/or fund endorsed proposals. |  |
| 40 | Maximizing benefits of research | Smart contract | Blockchain used to automate or manage innovation enhancing tools, such as a BrainSwarming tool; allowing pseudonymised editing (including to refinements, goals, and creation of new graphs); with embedded AI technology to suggest analogues; may include award of tokens for effective solution paths. | * |
|  | Maximizing benefits of treatment | Automation |  |  |
| 41 | Express/communicate preference/choice | Transparent register | Skill marketplace; automatically suggesting matches between 'data owners' (the data may be skills or actual data; who indicate their preference of data sharing) and 'data searchers' (such as researchers or others looking for individuals who hold certain data or skills). |  |
|  | Reducing waste | Transparent register |  |  |
| 42 | Express/communicate preference/choice | System of rules | DAO to organise a political interest group, potentially formed of scientists, healthcare workers, and patients. |  |
| 43 | Express/communicate preference/choice | Smart contract | DAO managed funding pool for innovative start-ups - funders buy tokens and vote on proposals, winner(s) by vote receive funding; funders receive proportional share of IP. | * |
| 44 | Improving replicability | Transparent register | Monetised pseudonymous post-publication peer-review. |  |
| 45 | Improving use of and access to protocols | Smart contract | Token payments for higher-quality protocols/studies. |  |
| 46 | Maximizing benefits of research | Smart contract | Token payments for allowing federated learning. |  |
| 47 | Maximizing benefits of research | Community | Blockchain managed seed funding, e.g. token-based funding rounds for research projects. |  |
| 48 | Maximizing benefits of research | Smart contract | Tokens in place of grant funding; use restricted to governance, research, or other relevant costs. | * |
| 49 | Rewarding contributions to research | Community | Practical implementation of Health Impact Fund. |  |
| 50 | Maximizing benefits of research | Smart contract | Token payments for successful improvements to eg. protocols. |  |
| 51 | Improving record keeping | Immutable audit trail | Tracking of health (including fitness) behaviour for research. |  |
| 52 | Improving equality of representation of research users | Smart contract | Token payments awarded for researchers who use representative samples in their studies. |  |
| 53 | Reducing waste | Smart contract | Token payments for spotting and/or fixing errors or problems, eg. within a registered protocol. |  |
| 54 | Reducing error, fraud, and waste | Encryption | Patients provide pseudonymised feedback and/or ratings which are publicly visible. |  |
| 55 | Rewards for contributions to research | Automation | DAO/smart contract which instantly rewards a percentage of savings to any entity demonstrating improvements in cost/time efficiency. |  |
| 56 | Reducing waste | Community | DAO to manage insurance and liability - 'insurance without the insurance company'. |  |
| 57 | Rewards for contributions to research | Shared interest | Rewards for specific social improvement achieved through research project, eg. green coins for climate emission reductions. |  |
| 58 | Improving record keeping | Automation | Automated analysis (may be statistical) of data uploaded from IoT for healthcare. |  |
| 59 | Desert | Immutable audit trail | Minting NFTs that represent ownership, which can be traded or fractionalised; complement or alternative to patent system. | * |
| 60 | Express/communicate preference/choice | Consensus mechanism | DAO-based voting as a method of determining scientific consensus. | * |
| 61 | Maximizing benefits of research | Immutable audit trail | NFTs distributed to individuals holding a personal dataset, for example those with a rare mutation, or whose genetic or proteomic sequence is used for research. |  |
|  | Improving record keeping | Immutable audit trail |  |  |
| 62 | Desert | Smart contract | Guaranteed sharing of patent profits in pre-agreed manner. |  |
| 63 | Desert | Smart contract | Enable partial authors and other contributors to be rewarded for participation in research via tokens; could be an incentive to assist. |  |
| 64 | Express/communicate preference/choice | Consensus mechanism | Enabling democratic voting on proposals, amendments, etc. |  |
| 65 | Improving record keeping | Immutable audit trail | NFTs distributed to individuals holding a personal dataset, in order to prevent access to unauthorised individuals. |  |
| 66 | Rewards for contributions to research | Shared interest | Trust tokens for healthcare professionals or researchers. |  |
| 67 | Reducing waste | Automation | DAO managed distribution of healthcare aid (funds). |  |
| 68 | Reducing waste | Transparent register | Labour exchange for medical or research staffing, including option for certifications, ratings, and pseudonymity. Individuals prosent depending on factors such as salary; exchange is automatically updated and distributed. | * |
| 69 | Reducing waste | Encryption | DAO managed pseudonymous recycling of used/excess reusable devices. |  |
| 70 | Express/communicate preference/choice | Transparent register | Practical implementation of meta-consent. | * |
| 71 | Improving record keeping | Automation | Tracking of health (including fitness) behaviour for treatment; may be incentivised by tokens. |  |
| 72 | Improving record keeping | Automation | Gamification: token payments for healthy lifestyle / behaviours. | * |
|  | Prevent harm | Smart contract |  |  |
| 73 | Rewards for contributions to research | Smart contract | Micropayments to authors / scholars when their work is accessed or viewed. | * |
| 74 | Maximizing benefits of research | Automation | Automatic data extraction and analysis from research DAOs. |  |
| 75 | Maximizing benefits of treatment | Smart contract | Token incentives for healthcare professionals to undertake CPD. |  |
| 76 | Maximizing benefits of treatment | Automation | Real-time update of data in online publications, may include journal articles. | * |
|  | Maximizing benefits of research | Automation |  |  |
| 77 | Improving record keeping | Automation | Quantified personal dense dynamic data clouds. |  |
| 78 | Reducing error | Automation | Automatic tracking of post-publication events - including replication attempts, errors, retrations, translations. |  |
| 79 | Reducing fraud | Automation | Automatic analysis of study data to prevent fraud such as made-up statistics. |  |
| 80 | Improved record keeping | Automation | Automated alert system if device output crosses warning threshold. |  |
| 81 | Improving review | Smart contract | Monetised pseudonymised peer-review. |  |
| 82 | Maximizing benefits of treatment | Smart contract | Token payments for teaching. |  |
| 83 | Improving use of and access to protocols | Smart contract | Token payment for executing according to protocol. |  |
| 84 | Improving use of and access to protocols | Automation | Automatic check and notification for the same or similar protocols. |  |
| 85 | Rewards for contributions to research | Smart contract | Citizen science: Token payments to citizens who make important scientific discoveries or progress; may include NFTs. |  |
| 86 | Improving replicability | Smart contract | Token payments to researchers who carry out replication studies. |  |
| 87 | Improved record keeping | Transparent register | On chain DOIs of all publications - automatically submitted to a repository / single database. |  |
| 88 | Reducing error | Automation | Automation of clinical audits. |  |
| 89 | Reducing fraud | Automation | Automated tamper-proof delivery of information to insurance companies. |  |
| 90 | Improving replicability | Transparent register | Registering choices made under 'researcher degrees of freedom' during trial registration or research process. |  |
| 91 | Improving use of and access to protocols | Timestamping | Timestamping of protocol phase completion. |  |
| 92 | Improving replicability | Timestamping | Timestamped verified snapshot of data at specific or random stage of research, automatically delivered to pre-specified interested parties eg. funders. | * |
| 93 | Increase efficiency of recruitment | Encryption | Publicly available pseudonymous record of patients for recruitment (for example, by disease, or by willingness to consent). |  |
| 94 | Desert | Transparent register | Registry of non-assignable rights, eg. for ghost writers. |  |
| 95 | Reducing disproportionate governance/ethical review | Transparent register | IRB decisions stored on blockchain, decision relating to paper made public on paper acceptance/publication. | * |
| 96 | Reducing fraud | Transparent register | Qualifications (eg. medical board certificates, research degrees) hashed and uploaded to blockchain to enable open verification. |  |
| 97 | Maximizing benefits of treatment | Transparent register | Blockchain for healthcare record management. |  |
| 98 | Reducing duplication | Automation | Smart contracts enable efficient search function for similar items. |  |
| 99 | Reducing fraud | Automation | Automated checks on suggested reviewers to exclude those eg. with known conflicts, from same organisation. | * |
| 100 | Improved record keeping | Encryption | Pseudonymised verified system to register suggestions for improvements in research teams, labs, hospitals, etc. |  |
